# What We Know about the Public’s Level of Concern for Farm Animal Welfare in Food Production in Developed Countries

**DOI:** 10.3390/ani6110074

**Published:** 2016-11-16

**Authors:** Amelia Cornish, David Raubenheimer, Paul McGreevy

**Affiliations:** 1 Faculty of Veterinary Science, University of Sydney, Sydney, NSW 2006, Australia; paul.mcgreevy@sydney.edu.au; 2 Charles Perkins Centre and School of Life and Environmental Sciences, University of Sydney, Sydney, NSW 2006, Australia; david.raubenheimer@sydney.edu.au

**Keywords:** production animal welfare, animal sentience, concern for animal welfare, public knowledge

## Abstract

**Simple Summary:**

The production of food from animals poses many ethical challenges. This review explores what we know about different levels of concern for animal welfare in food production by such stakeholders as veterinarians, farmers, and the general public. Despite the general public’s level of concern for animal welfare in food production being high, their understanding and knowledge is poor. Thus, it is suggested that through widespread consciousness raising we can encourage the public to accurately translate their concerns into market drivers, in turn improving the welfare of billions of animals.

**Abstract:**

Population growth and rising consumption of meat, dairy, eggs and fish are forcing the world to face the intersecting challenges of how to sustainably feed a population expected to exceed 9 billion by 2050, while also controlling the impact of food production on the planet, on people and on animals. This review acknowledges the absence of a globally accepted definition of animal welfare and then explores the literature regarding different levels of concern for animal welfare in food production by such stakeholders as veterinarians, farmers, and the general public. It focuses on the evidence that the general public’s level of concern for animal welfare is linked to various demographic and personal characteristics, such as age, gender, religion, location, meat eating, and knowledge of animal welfare. Certain animals have characteristics that influence concern for their welfare, with those species that are considered more intelligent being afforded more concern. There is compelling evidence that the general public’s understanding of animal welfare in food production is poor. Acknowledging that public concern can be a driving force to change current production methods, the authors suggest widespread consciousness raising to redefine socially acceptable methods of food production from animals and to ensure that it remains in step with societal concerns.

## 1. Introduction

Human use of animals can take a variety of forms. By far the largest numbers of animals in the human domain are used in food production, with total annual figures estimated at more than 70 billion animals [1]. With global population predicted to exceed 9 billion by 2050, food production is predicted to need to increase by 70% to 100% by 2050 [2,3]. To keep up with such demand, animal agriculture has moved in developed countries away from the predominantly small family farms that existed before the second agricultural revolution, and has become increasingly intensive. Such intensification is arguably producing more and cheaper food to feed a growing human population. However, it has also resulted in many unsustainable and deleterious environmental, social, human health and animal welfare issues [2,4].

The extent to which animals suffer in modern farming presents an ethical dilemma for consumers and producers alike. Despite its current popularity, public concern for animal welfare is neither new, nor likely to dwindle. The past two decades have seen public concern for animal welfare continue to rise [5,6], and today, animal protection legislation exists across many countries [7]. Our knowledge and understanding of animal welfare and sentience underpin the societal concerns that call for such legislation [8,9]. The current review aims to address what is already known in the literature regarding the public’s concern, as well as that of veterinarians’ and farmers’, for the welfare of animals used in food production. The authors highlight the literature that suggests the public have only a rudimentary understanding of animal welfare in food production [10]. To improve the welfare of the billions of animals farmed for food annually, we must first address the shortcomings in the public’s understanding, knowledge and awareness of the environmental, social, human health and animal welfare impacts of all animal production systems, particularly modern intensive production systems. In turn, consumers can be a driving force to raise the current standards of farm animal welfare by accurately translating their preferences and concerns into market drivers.

## 2. What Is Animal Welfare and How Do We Measure It?

To understand public concern for animal welfare, we must begin by asking *What is animal welfare?* Historically, veterinarians and farmers primarily understood animal welfare in terms of animal health and productivity, and they paid less regard to animals’ feelings and mental states [11], but the past few decades have seen a rapid development in scientific approaches to the study of animal welfare [12]. One of the first major public expressions of concern for the welfare of animals, particularly regarding the then-new production system of confinement, was sparked by Ruth Harrison’s book *Animal Machines* (1964), which exposed the welfare issues in intensive farming [13,14]. Harrison’s book prompted the British government to commission the 1965 investigation, led by Professor Roger Brambell, into the welfare of intensively farmed animals. Since that investigation, the idea of the *Five Freedoms* was posited by the UK’s Farm Animal Welfare Council (FAWC) in 1979. Since then, the Five Freedoms have become internationally recognised as a statement of fundamental principles of animal welfare and continue to provide a valuable framework to measure animal welfare [15].

The concept of the *Five Domains of Potential Welfare Compromise* (the Five Domains) was formulated in 1994 by Mellor and Reid [16], and a recent review and updated thinking can be found in a paper by Mellor [17]. The domains expand the Five Freedoms, but express welfare in terms of compromise rather than freedoms. The first four Domains focus on physical/functional welfare and are Nutrition, Environment, Health and Behaviour. The last represents the experience of the animal, known as the Mental State [16,17].

In the European Union (EU), public concern about animal welfare and the findings that consumers do not feel they are provided with adequate information about the welfare of farmed animals, led the EU to fund the development of the Welfare Quality project in 1994. The project aimed to develop evidence-based tools for assessing animal welfare on farms and at slaughterhouses, to identify the main animal welfare problems, and to inform welfare improvement strategies. The Welfare Quality approach has identified 12 criteria and four principles that summarise the main areas of concern needing to be assessed [18].

Animal welfare is a multi-dimensional concept [19]. Scientists have debated what the direction of animal welfare research should be, with many proposing different definitions, research methods and means of interpreting welfare [20,21]. Currently, the World Organisation for Animal Health (OIE) is the primary international standard-setting organisation for veterinary concerns and it provides guidelines, codes and science-based standards for various aspects of animal health to member states [15,22]. The OIE ensures that animal welfare is an international priority through the OIE Terrestrial Animal Health Code (Terrestrial Code). The OIE defines animal welfare as “how an animal is coping with the conditions in which it lives. An animal is in a good state of welfare if (as indicated by scientific evidence) it is healthy, comfortable, well nourished, safe, able to express innate behaviour, and if it is not suffering from unpleasant states such as pain, fear, and distress” [22].

Currently, the three main scientific approaches to animal welfare most widely used to define and assess the status of an animal’s welfare are biological function, affective state, and natural living [23]. The first of these approaches came about in the early 1980s and emphasised the biological functioning of organisms, such as in reproduction, with good welfare requiring the absence of stress [24]. In the early 1990s the second approach arose and focused on the psychological aspects of welfare and the mental experiences of animals. By the early 2000s, this proposition had become widely accepted and today it is the basis of much animal welfare science discourse [25]. The third, most recent approach highlights the need for animals to live naturally and to have the ability to express innate behaviours [23].

As animal welfare science has evolved, the acceptance of mental experiences as representing welfare has increased in importance [26]. The concept of *Quality of Life* (QoL) was developed to provide a multifactorial, comprehensive approach to assess an animal’s mental well-being [26]. Although QoL assessments can give rise to empathetic speculation and anthropomorphic projections, such issues can be minimised through the use of objectively based methodologies [26]. Toward this end, the FAWC refined the QoL concept by including categories of *a life not worth living*, *a life worth living* and *a good life*. This enabled QoL assessments to be made using a scale that ranges from the worst possible life, to a life that is neither good nor bad through to the best possible life [26]. Subsequently, Yeates added the concept of *a life worth avoiding* [27], which highlights the role of veterinary intervention when negative experiences outweigh positive experiences.

Nowadays, scientific understanding of animal welfare has evolved beyond simply the absence of suffering to include the quality of an animal’s entire experience with its environment, such as positive states, preferences, motivations and aversions [11,13,25,26]. Despite, or perhaps even because of these advances, there is no simple way to measure an animal’s welfare. For researchers to assess animal welfare requires a number of assumptions, mainly due to differences in measurements, welfare criteria and welfare interpretations [28]. Nonetheless, the importance of measuring animal welfare is critical to any effort to identify those farm systems that provide the highest levels of animal welfare [12]. The animal welfare science literature proposes several ways to measure animal welfare, including physiological and productivity measures, and behavioural responses [12,29]. These are assessed, inter alia, using health measures such as skin lesions [30], productivity traits, such as milk yield [31], and physiological stress responses, such as heart rate [32].

## 3. The Animal Sentience Debate That Underpins Public Concern for Animal Welfare

The science of animal sentience underpins the entire animal protection movement [9] and identifies which animals are likely to feel suffering and should thus be protected [29,33]. Animal sentience refers to the notion that an animal experiences not just pain but positive and negative emotions, such as joy and pleasure [34]. As with *animal welfare* there is also a lack of consensus around a globally accepted definition for *animal sentience* [35]. Nonetheless, if farm animals are sentient, there are significant implications for the ethics of food production.

Debate regarding whether animals are sentient beings dates back many centuries [8,9]. For example, Descartes, the 17th century French philosopher, viewed animals as nothing more than automata, incapable of feeling or suffering [8], whereas evolutionary theorist Charles Darwin posited that animals are capable of self-consciousness [36], and his research, from an evolutionary fitness perspective, validated the concept of sentience [36,37]. Animal sentience was also supported by ethologists such as Nicol and Guilford, and Dawkins; whose pioneering research into motivational studies in animals assumed that the effort an animal is prepared to undertake to achieve or avoid a stimulus provides a proxy for the positive or negative experiences the animal experiences when exposed to the stimulus [29,38,39].

Despite such advancements, scientific progress regarding animal sentience has often been hindered by our inability to reliably measure it [9,29]. Nonetheless, today there is widespread recognition of animal sentience, particularly in vertebrate species [33], and animals were acknowledged as sentient beings in the *Amsterdam Treaty* in 1997. The *Amsterdam Treaty* confers special consideration for animals under European Law [40]. Additionally, in 2012, a group of prominent international scientists signed the *Cambridge Declaration on Consciousness*, asserting their support for the idea that animals possess consciousness and are aware to the degree that humans are [41]. However, there remains debate regarding sentience in fish as they lack the complex neuroanatomical structures found to be associated with conscious subjective states in humans [9,42,43]. Examples of complex cognitive abilities in fish have been offered as evidence of their neurological and physiological ability to experience subjective states [44], yet critics suggest that such responses merely reveal unconscious reflexive pathways [42,43].

Despite their huge numbers, the welfare of invertebrates is often overlooked when compared to vertebrates due to controversy surrounding invertebrate sentience and the assumption that these animals are incapable of feeling pain and suffering [45], but scientific reports continue to reveal behavioural indicators that could infer sentience in invertebrates. These include pain responses by cephalopod molluscs [45] and crabs [46], complex reproductive behaviour by crayfish [47], and pessimistic cognitive bias as a measure of negative emotional states in honeybees [48].

## 4. Veterinarians’ and Veterinary Students’ Level of Concern for Animal Welfare

Veterinarians play a fundamental role in the promotion and safeguarding of animal welfare [49,50]. Despite this critical role, they often have overlapping and sometimes conflicting responsibilities to the animals in their care, to their clients, employers and the general public [51]. The literature reveals gender to be a consistent predictor of veterinarians’ level of concern for animal welfare, with female veterinarians and veterinary students showing more concern for animal welfare than males [52,53].

Much of the animal welfare science literature explores the attitudes of veterinarians and veterinary students to pain in animals [53,54,55,56,57]. A survey of veterinarians in New Zealand found that females scored animal pain at higher levels than their male colleagues did, implying that female veterinarians were more sensitive to the possibility that animals experience pain, and showed more empathy toward animal pain [53]. A Canadian study found that pain treatment use by veterinarians was inadequate, and that they often did not give pain treatment to young animals, particularly at the time of castration [56]. Furthermore, less than half of livestock veterinarians in the UK reported that they had sufficient knowledge of cattle pain assessment and treatment [57]. Similarly, 42% of small animal veterinarians in New Zealand reported inadequate knowledge of animal pain assessment and treatment [53]. Several studies have explored the reasons given by veterinarians for their poor use of pain treatment. These include anaesthesia and analgesia being too time consuming, doubt about the general public’s willingness to support the additional costs involved, difficulty in recognising pain in animals, concern for human food safety, the lack of long-acting, cost-effective analgesics available for livestock, and the long or unknown withdrawal periods for opioids and dissociative anesthetics in farmed animals [54,55,56].

There is evidence that veterinarians’ and veterinary students’ concerns for animal welfare vary among species. Veterinary students at Cornell University in the US were found more likely to agree that dogs and cats had cognitive abilities as opposed to farm animals, as well as being more likely to consider certain agricultural procedures humane for farm animals but not for dogs and cats [58]. Additionally, pain ratings and pain treatment use by veterinarians were found to vary among species, with more consideration being given to horses than to cows and pigs [56]. Furthermore, a US study found that veterinary college faculty members and animal science faculty members considered current production methods provided adequate welfare for beef, sheep and dairy, but not for pigs and poultry [59].

The literature suggests relationships between stage-of-study in the veterinary course and students’ attitudes to animal welfare, with students in their earlier years assigning higher levels of sentience to animals than those in later years [52]. A US study found that fourth-year students were less likely than second- or third-year students to treat animals for pain, suggesting that students’ perceptions of pain in animals decreased as they progressed in their courses [60]. Similarly, sensitivity to the role of the human–animal bond has been found to decrease as students progressed in their studies [61]. Despite the worrying implications of such findings, interventions such as an animal welfare and ethics course [62], and workplace learning [63] have been found to improve the level of concern and empathy for animals shown by students.

## 5. Farmers’ Level of Concern for Animal Welfare

Farmers are the caregivers of animals and so are a pivotal influencer of animal welfare, behaviour, health, and production [64,65,66]. Several studies have examined farmers’ attitudes to animal welfare and the impact of specific welfare improvement measures on farm animals [30,64,65,66,67]. Farmers’ attitudes are strong, consistent predictors of their behaviours toward their animals and the importance they place on the human–animal relationship [31], as well as the stress, productivity and human-related fear of the farm animals themselves [65,67].

Studies have revealed discordance between the publics’ and farmers’ perceptions of animal welfare [68,69]. In general, the public was concerned with the welfare of farm animals that have a compromised ability to express natural behaviours and whether they have enough space to move freely [55,68], while farmers were chiefly concerned with the physical condition and productivity of animals rather than their behavioural and/or social needs [66,68]. Moreover, farmers were found to perceive the welfare of farmed animals as positive or satisfactory, whereas consumers reported overall negative perceptions [68,69].

Farmers’ perceptions of animal welfare can be grouped into two main categories: those who view animal welfare standards as a means of achieving economic results; and those who view those standards as a way of satisfying moral and ethical considerations in animal production [69,70,71]. Studies suggest that farmers’ concerns for animal welfare vary according to farming techniques and whether respondents are engaged in third-party schemes [70,71]. For example, European farmers engaged in organic or specific animal welfare schemes were more concerned with animals’ ability to express natural behaviours, while farmers engaged in a quality-assurance scheme (for example, food safety or traceability schemes) were more concerned about animal health and productivity [71].

Farmers’ attitudes to positive human–animal interactions have been found to influence their behaviours and positively affect animal welfare and productivity [30,31,65,66,67]. Positive human–animal relationships, such as stroking of animals, have been shown by many studies to promote positive affective states in animals and to improve production [31,65,72]. Conversely, farmers who used aversive handling techniques, such as pushing, were associated with poorer animal welfare and reduced meat quality [64,65]. Bertenshaw and Rowlinson [31], for example, found that milk yield per lactation was 258 L higher on farms where cows were called by individual names. Given the abundant evidence that farmers’ attitudes and behaviours influence the welfare, health, behaviour and productivity of animals, studies have examined the role of training programs to modify farmers’ attitudes and behaviours with promising results for animal welfare [73,74].

## 6. The Public’s Knowledge of Animal Welfare Issues in Food Production

Understanding public concern for animal welfare must begin with understanding the role of knowledge in shaping attitudes toward animal welfare. In the EU, knowledge about animal welfare was found to be a stronger indicator of views on the importance of animal welfare than social or demographic factors [75]. Today, food consumption is often separated from food production, and the general public has very little, if any, direct experience with farmers, production animals or slaughterhouses. As a result, consumers seem confused and misinformed about farm animal welfare issues and farming practices [76]. Furthermore, many consumers actively avoid learning about the conditions imposed on farm animals or remain in willful ignorance (e.g., dissociating meat from its animal origin) to avoid the realities of food production [10,77,78,79].

In the EU, knowledge of production animal welfare was found to be partly associated with age and education. For example, respondents educated beyond the age of 20 were the most likely (76%) to report at least some knowledge of animal welfare. Respondents who reported that they were knowledgeable about farm animal welfare and believed it needed to be improved were more likely to see animal welfare as an important issue [75]. This is confirmed in the literature, for example, by a US study at Michigan State University, allowing for multiple responses, found that 40% of introductory animal science students and 70% of applied animal behaviour students showed some concern for how animals in intensive production systems were treated. Most interestingly, the animal science students scored below chance performance and the animal behaviour students scored above 80% in identifying cages as the main housing for laying chickens [80], inferring a link between concern and knowledge. There has also been found to be a strong association between extant knowledge about animal welfare and wanting to know more [75]. Recently, consumers’ desires for production animal welfare information have increased across the EU [81]. Similarly, a Canadian study reported that most respondents expressed a desire for additional knowledge regarding animal production practices [82].

Again in the EU, 69% of respondents claimed to have “some” knowledge of animal farming in their country, 57% claimed to know “a little”, 28% claimed to know “nothing at all” and only 12% claimed to have “a lot” of knowledge. Additionally, 54% agreed that it was not easy to find information on animal welfare when shopping and 58% stated that they would like more information [75]. Likewise, 44%, 39% and 45% of consumers in Italy, Great Britain and Sweden, respectively, *disagreed* with the statement that “I feel sufficiently well-informed about animal welfare” [83]. Similarly, Hall et al. [84] found that most UK respondents admitted that they knew very little about how meat chickens were farmed.

Research suggests that knowledge of animal welfare issues is often based on personal experience with animals, the media, animal advocacy groups, magazines, radio, newspapers, friends and family. An EU study found that television was the most cited source of information, followed by the internet and newspapers [75], with most animal welfare information coming from mass media images and reports, particularly when reporting focused predominantly on negative issues (e.g., bovine spongiform encephalopathy (BSE) or *Salmonella* outbreaks) [79,85]. Recently, an online survey of almost 800 US households found more than half (56%) of respondents could not cite a source for animal welfare information, and those who could were found to obtain information from animal protection organisations, such as People for the Ethical Treatment of Animals (PETA) [86]. Tonsor and Olynk [87] found a clear link between negative animal welfare information and a decline in demand for meat across all livestock industries. Such findings were supported by McKendree et al. [86], who found that recent media attention around the US swine industry had resulted in 14% of respondents reducing their pork consumption by an average of more than 50%, due to animal welfare concerns.

## 7. The Public’s Level of Concern for Animal Welfare

Public concern for animal welfare is rising [5,6]. Research suggests positive attitudes to animals are associated with higher levels of concern and empathy for animal welfare [88,89]. As mentioned above, studies from the EU found knowledge to be a stronger indicator of concern than social or demographic components [75]. However, findings regarding egg-purchasing preferences in British Columbia found that consumer preference for eggs differed significantly with demographics. Caged-egg consumers were less concerned about animal welfare, less educated, older, more sensitive to price, and shopped in major chain retailers. Non-caged-egg consumers were more concerned about animal welfare, less price sensitive, and shopped at farmers’ markets, and local or organic-grocery retailers [90].

Consumer concern for animal welfare is well documented in the literature. An Australian study found that 71% agreed with the statement “farm animal welfare is an important consideration” [91]. Similarly, 68% of respondents in Scotland were “concerned” (48%) or “strongly concerned” (20%) [6], and 86% of Dutch respondents were either “somewhat concerned” (45%) or “very concerned” (41%) about production animal welfare [92]. Additionally, 74% of respondents surveyed in the EU believed they could influence animal welfare conditions by purchasing welfare-friendly products [93]. Moreover, when purchasing behaviour was surveyed directly, 43% of EU consumers reported that they considered animal welfare when buying meat, with 34% reporting that animal welfare was of the highest importance in contrast to only 2% who reported that it was not important at all [75].

Consumers have been found to provide qualified support for intensive animal farming, and not to hold farmers accountable for animal suffering in food production [68]. Instead, consumers have been found to criticise profit-driven industry and the growing consumer demand for cheap food without thinking about the animal welfare implications [82]. The general public has also been found to be cynical about their ability as consumers to improve animal welfare and, as a result, to abrogate responsibility for farm animal welfare to other agents, such as governments [77].

## 8. Age and the Public’s Level of Concern for Animal Welfare

Research reveals a negative correlation between public concern for animals and age [94,95,96]. Some scholars suggest that the influence of age is a cohort effect due to the impact of a shared history [97], and others suggest it is due to mental attributes becoming more complex with age [98]. Jamieson et al. [99] found that despite having only limited knowledge of farm animal welfare problems, UK adolescents were concerned about farm animal welfare, but did not believe they had any power to elicit positive changes for animals, and most often assigned responsibility for farm animal welfare to governments and farmers. Nonetheless, younger people have been found to show more concern for animal use and to be more engaged in animal welfare issues than older people [94,95]. Older people were found to have a more utilitarian view of animals and to highlight the practical and material value of animals, compared to children, who held a more naturalistic view [95]. Younger people have higher levels of concern for animal welfare, yet they are less concerned for farm animals than companion animals and use distancing mechanisms to accept human use of animals [99].

## 9. Gender and the Public’s Level of Concern for Animal Welfare

Gender is a consistent predictor of concern for animals, with women showing more concern [52,94,96,97,100,101] and being more likely to support the animal protection movement [100]. Women hold more negative attitudes toward human use of animals [96] and were more strongly opposed to animal experimentation [100,101]. Women were also found to attach more importance to farm animal welfare relative to other product characteristics and to consider the current state of animal welfare as poor [102]. Two studies of veterinary students found that female students considered the role of the human–animal bond to be more important in their lives and that they held stronger beliefs in an animal’s capacity to experience emotions, such as pain and hunger, than male students [52,61].

Baron-Cohen [98] suggests that, due to hormonal and genetic differences between the sexes, women are more likely to empathise spontaneously, while males are more likely to systematise spontaneously and are less likely to empathise. However, other scholars have identified that the role of gender in concern for animal welfare is not necessarily as simple as the opinions of women versus those of men. Some studies have postulated that it is the masculine and feminine dimensions of sex-role orientation, rather than gender, that relates to concern for animals [103,104]. For example, Herzog et al. [103] found that people who identify as feminine rather than masculine showed greater concern for animal welfare. Furthermore, Peek et al. [104] proposed that it is women’s social role and structural location in society that accounts for their elevated concern for animals. Similarly, Kendall et al. [97] argued that women are usually the primary caregivers for families and responsible for household tasks that put them in contact with animals, such as feeding companion animals. Such findings suggest the way females are perceived or perceive themselves in a household or society can influence their concern for animal welfare and their ability to voice their opinions and concerns. Additionally, when looking at the role of men or masculinity in society, historically men were responsible for hunting, while women gathered fruits and vegetables. Studies have found that hunting is associated with less concern for animal welfare [97] and Kellert (1996) as cited in [97] found that men are more likely to engage in activities such as hunting. Also, in many modern societies meat, especially red meat, is a somewhat masculine food and men are often responsible for activities such as barbequing meat or carving roasts [105]. Thus, such findings could also account for some degree of the gender difference shown between men and women in concern for animal welfare [97,105].

Additionally, there is a gender difference evident in the way people justify eating meat [105,106]. Adams’ 1990 *The Sexual Politics of Meat* theory links meat eating with masculinity, patriarchy and power, while vegetarianism is linked with femininity [105]. A study of undergraduate university students found that males were likely to eat more meat than females and used direct strategies to justify eating meat, such as endorsing pro-meat attitudes and denying animals’ ability to suffer. Females ate less meat and were more apologetic about eating meat and used indirect strategies, such as dissociating animals from food [106].

## 10. Religion and the Public’s Level of Concern for Animal Welfare

Religion has been found to influence concerns and attitudes towards animals [55,94,107,108]. A US study of animal science faculty members found a negative correlation between a higher sense of religiosity and concern for farm animal welfare [55]. Christianity was shown to be positively associated with support for animal use in research. For example, Bowd and Bowd [107] surveyed people from five Christian denominations, spanning liberal to conservative groupings, and found the more liberal denominations generally held more humane attitudes to animals. Driscoll [94] found that people’s views differed across Christian denominations, with people who identified no religious affiliation or an affiliation with the Catholic Church considering animal use in research to be less acceptable than those who identified with a traditional Protestant affiliation. Similarly, attending a church of any denomination has been linked to less concern for animal welfare [108]. Furthermore, it is worth noting that other religions place different consideration on certain animal species that may affect people’s level of concern for those species, such as cows being respected in Hinduism and pig meat being avoided in Judaism.

## 11. Experiences with Animals and the Public’s Level of Concern for Animal Welfare

Research has found concern for animal welfare is influenced by past experiences and familiarity with animals, for example, owning a companion animal [10,94,97,109]. Childhood experience has been shown to have the greatest place-based effect on attitudes to animals [97]. People who had a companion animal were found to be more likely to rate animal research as less acceptable [94], more likely to be vegetarian or vegan, and more likely to support animal welfare groups [109]. Such findings support Allport’s *Contact Hypothesis* (1954) that assumes contact and experience with members of another group (e.g., non-human animals) leads to a better understanding, and fosters stronger emotional attachment and empathy to them [110]. Furthermore, whether or not people had visited a farm affected their perceptions of farm animals and level of concern for farm animal welfare. For example, UK students who had previously visited a conventional working pig farm showed increased concern for farm animal welfare and a willingness to pay more for products when improved welfare [111] was the factor increasing price.

## 12. Rural or Urban Living and the Public’s Level of Concern for Animal Welfare

Rural and urban dwellers have been found to vary in their level of concern for animal welfare [6,97], indicating that living circumstances provide different experiences and relationships with animals that affect people’s attitudes to them [97]. Country people were found to show less concern for farm animal welfare compared to their urban counterparts [97,102]. Furthermore, when exploring people’s level of knowledge regarding farm animal welfare and production systems, rural residents were found to have better farm knowledge and experience, while urban dwellers had poorer knowledge regarding livestock [79].

## 13. Speciesism and the Public’s Level of Concern for Animal Welfare

Peter Singer’s book *Animal Liberation*, first published in 1975, discussed the concept of speciesism and the notion that being human is a good enough reason for deserving greater moral rights than non-human animals [112]. Today, the debate continues, with Kagan’s article *What’s Wrong with Speciesism?* (a critique of Singer’s claims [113]), and Singer’s subsequent response*Why Speciesism is Wrong: A Response to Kagan* [114]. Nonetheless, evidence suggests that people discriminate among non-human animal species, with much of human concern for animal welfare and acceptance of animal sentience being linked to the perception of an animal’s position on the phylogenetic scale relative to humans [78,88,94,115]. Belief in animal minds (BAM) refers to ascribing mental capacities, such as intellect and emotions, to animals. BAM has been found to be a stronger predictor of disapproval of animal use than demographic variables, such as age and gender [96]. Similarly, people’s concerns for animal welfare are affected by their perceptions of an animal’s cognitive abilities, such as intelligence [10,88]. Knight and Barnett [10] found UK respondents based their perceptions regarding animal use for certain purposes on their perception of the mental capacity of the animals, as well as their familiarity and past experience with those species.

A study from the US asked respondents to rate 33 species in terms of intelligence and lovability. It found that the highest rated were primates and larger mammals; the lowest rated were spiders, insects and some mammals. Food animals, such as chickens, lobsters and trout, were rated the lowest on intelligence and rated next-to-lowest on lovability [116]. A similar study asked students from several different countries to rate the order of sentience of several common species and found that in descending order the rankings were monkey, dog, newborn human baby, fox, pig, chicken, rat and fish [115]. The study also found that students’ acceptance of animal sentience was related to their attitudes to animal use. For example, students who assigned chickens higher levels of sentience were also found to disagree with the statement “as long as adequate food, warmth and light are provided, there is nothing really cruel about battery-hen farming” [115]. Recently, a novel Australian study that involved the use of chickens to teach animal training skills to undergraduate students found that the students’ attitudes to sentience in animals improved. The experience also improved perception of chickens’ intelligence and capacity to experience affective states [88].

## 14. Meat Eating and the Public’s Level of Concern for Animal Welfare

Eating meat poses a moral paradox for many people [117,118,119]. Studies have identified socio-demographic variations among meat consumers, with women and those who are highly educated, with higher incomes, and living in urban locations found to eat less meat and to purchase more welfare-friendly products [102,106,120]. People who enjoy eating meat were found to be more masculine [105,106,119] and showed less moral concern for animals [119]. Moreover, people reported to be against animal experimentation were more often vegetarian or supportive of vegetarianism [101]. Additionally, an EU study found that vegetarians were more concerned about animal well-being and donated more often to animal-oriented charities [121]. Bastian et al. [117] found an inverse relationship between the attribution of mental abilities to certain species (e.g., cows and chickens) and their edibility rating. Furthermore, Prunty and Apple [118] found US non-vegetarian university students showed more concern for animal welfare and were open to eating less meat after exposure to the animal welfare statement: “animals should not suffer needlessly in the production of meat”, implying that students realigned their attitudes to resolve the self-contradiction between their attitudes and behaviours.

According to Festinger’s theory of cognitive dissonance, the aversive affective state that arises when consumers are conflicted between enjoying eating meat and being concerned for animal welfare generally demands resolution [122]. For example, Loughnan et al. [119] asked UK respondents to rate their level of moral concern for animals, in particular cows, while eating dried beef or dried nuts. The study found that the dried beef eaters showed lower moral concern for animals and rated the moral status of cows lower than the nut eaters did. Similarly, Australian respondents were first asked to rate their belief in the mental capacities of certain animals before taking a test, and then asked to rate it again while eating a meat product from the same animals, while at the same time being assessed using the daily mood scale—a measure of both positive and negative affect [117]. The results showed that respondents who lowered their animal mental capacity ratings while eating meat showed no negative affect, while the respondents who maintained consistent scores continued to endure a negative affective state [117].

## 15. Conclusions 

Despite the discordance between the attitudes and levels of concern shown by the general public, veterinarians and farmers, there is evidence of promisingly high levels of public concern for animal welfare across many developed countries. Public concern can be a driving force to change current production methods. Consumers have the power to raise the standards of farm animal welfare by accurately translating their preferences and concerns into accurate market drivers and market signals. In turn, farmers may be motivated to change their practice to meet consumer expectations. In addition, highlighting the performance and productivity benefits of better animal welfare can further encourage farmers to improve current production methods.

There is evidence that the general public’s level of concern for animal welfare is linked to demographic and personal characteristics, such as age, gender, religion, urban or rural locations, as well as perceptions of the intelligence and cognitive abilities of certain species, with animals considered to be more intelligent and closer to humans often being afforded more concern. However, despite such demographic and social influences, the literature suggests that knowledge is an even stronger influence on concern for animal welfare. There is evidence of an existing widespread knowledge gap regarding animal welfare in food production.

Knowledge has been shown to play a fundamental role in influencing and underpinning concern for animal welfare. The literature suggests that self-reported knowledge of farm animal welfare issues and exposure to farm animals, for example through direct agricultural experience, were linked with heightened levels of concern for animal welfare and more welfare-friendly behaviours. Today, modern consumers face the issue of food production often being removed from consumption. As a result, people have poor knowledge and understanding of animal welfare issues in animal production, particularly, modern intensive food production. However, animal welfare outcomes are not as simple as a consideration of extensive versus intensive systems. Many modern extensive systems can result in lower welfare. Thus, there is a greater need than ever before for public education and consciousness raising regarding the environmental, social, human health and animal welfare impacts of all animal production systems. By improving public knowledge, awareness and understanding of animal welfare in food production, we can elevate knowledge to align with current societal concerns, thus redefining socially acceptable methods of food production and improving the lives of the billions of animals farmed for food annually.

## References

[1] Compassion In World Farming (CIWF) (2013). Strategic Plan 2013–2017 for Kinder, Fairer Farming Worldwide.

[2] Gerber P.J., Steinfeld H., Henderson B., Mottet A., Opio C., Dijkman J., Falcucci A., Tempio G. (2013). Tackling Climate Change through Livestock—A Global Assessment of Emissions and Mitigation Opportunities.

[3] Godfray H.C., Beddington J.R., Crute I.R., Haddad L., Lawrence D., Muir J.F., Pretty J., Robinson S., Thomas S.M., Toulmin C. (2010). Food security: The challenge of feeding 9 billion people. Science.

[4] Steinfeld H. (2006). Livestock’s Long Shadow: Environmental Issues and Options.

[5] Bennett R.M., Blaney R.J. (2003). Estimating the benefits of farm animal legislation using the contingent valuation method. Agric. Econ..

[6] McEachern M.G., Schröder M.J., Willock J., Whitelock J., Mason R. (2007). Exploring ethical brand extensions and consumer buying behaviour: The RSPCA and the “Freedom Food” brand. JPBM.

[7] Barnett J.L. (2007). Effects of confinement and research needs to underpin welfare standards. J. Vet. Behav..

[8] Duncan I.J. (2006). The changing concept of animal sentience. Appl. Anim. Behav. Sci..

[9] Proctor H. (2012). Animal Sentience: Where are we and where are we heading?. Animals.

[10] Knight S., Barnett L. (2008). Justifying attitudes towards animal use: A qualitative study of people’s views and beliefs. Anthrozoös.

[11] Hewson C.J. (2003). What is animal welfare? Common definitions and their practical consequences. Can. Vet. J..

[12] Broom D.M. (2011). A History of Animal Welfare Science. Acta Biotheor..

[13] Fraser D. (2008). Understanding Animal Welfare: The Science in Its Cultural context.

[14] Harrison R. (1964). Animal Machines: The New Factory Farming Industry.

[15] Vapnek J.C., Chapman M. (2010). Legislative and Regulatory Options for Animal Welfare.

[16] Mellor D.J., Reid C.S., Baker R., Jenkin G., Mellor D.J. (1994). Concepts of animal well-being and predicting the impact of procedures on experimental animals. Improving the Well-Being of Animals in the Research Environment.

[17] Mellor D.J. (2016). Updating animal welfare thinking: Moving beyond the “Five Freedoms” towards “A Life Worth Living”. Animals.

[18] Blokhuis H.J. (2008). International cooperation in animal welfare: The Welfare Quality^®^ project. Acta Vet. Scand..

[19] Botreau R., Veisser A., Butterworth A., Bracke M.B., Keeling L.J. (2007). Definition of criteria for overall assessment of animal welfare. Anim. Welf..

[20] Duncan I.J., Fraser D., Appleby M., Hughes B.O. (1997). Understanding animal welfare. Animal Welfare.

[21] Mellor D.J., Patterson-Kane E., Stafford K.J. (2009). The Sciences of Animal Welfare.

[22] World Organisation for Animal Health (OIE) (2015). Terrestrial Animal Health Code.

[23] Fraser D. (2003). Assessing animal welfare at the farm and group level: The interplay of science and values. Anim. Welf..

[24] Broom D.M. (1991). Animal welfare: Concepts and measurement. J. Anim. Sci..

[25] Fraser D., Duncan I.J. (1998). ‘Pleasures’, ‘Pains’ and animal welfare: Toward a natural history of affect. Anim. Welf..

[26] Green T.C., Mellor D.J. (2011). Extending ideas about animal welfare assessment to include ‘quality of life’ and related concepts. N. Z. Vet. J..

[27] Yeates J.W. (2011). Is ‘a life worth living’ a concept worth having?. Anim. Welf..

[28] Mason G., Mendl M. (1993). Why is there no simple way of measuring animal welfare?. Anim. Welf..

[29] Dawkins M.S. (2006). Through animal eyes: What behaviour tells us. Appl. Anim. Behav. Sci..

[30] Kielland C., Skjerve E., Østerås O., Zanella A.J. (2010). Dairy farmer attitudes and empathy toward animals are associated with animal welfare indicators. J. Dairy Sci..

[31] Bertenshaw C.E., Rowlinson P.R. (2009). Exploring Stock Managers’ Perceptions of the human-animal relationship on dairy farms and an association with milk production. Anthrozoös.

[32] Rushen J., Munksgaard L., Marnet P.G., DePassille A.M. (2001). Human contact and the effects of acute stress on cows at milking. Appl. Anim. Behav. Sci..

[33] Broom D.M. (2007). Cognitive ability and sentience: Which aquatic animals should be protected?. Dis. Aquat. Org..

[34] Webster J. (2005). Animal Welfare: Limping towards Eden.

[35] Proctor H., Carder G., Cornish A. (2013). Searching for animal sentience: A systematic review of the scientific literature. Animals.

[36] Darwin C. (1872). The Expression of Emotions in Animals and Man.

[37] Webster J. (2006). Animal sentience and animal welfare: What is it to them and what is it to us?. Appl. Anim. Behav. Sci..

[38] Dawkins M.S. (1990). From an animal’s point of view: Motivation, fitness and animal welfare. Behav. Brain Sci..

[39] Nicol C.J., Guilford T. (1991). Exploratory activity as a measure of motivation in deprived hens. Anim. Behav..

[40] Webster J. (2013). Animal Husbandry Regained: The Place of Farm Animals in Sustainable Agriculture.

[41] Low P., Panksepp J., Reiss D., Edelman D., Van Swinderen B. The Cambridge declaration on consciousness. Presented at the Francis Crick Memorial Conference on Consciousness in Human and Non-Human Animals.

[42] Key B. (2016). Why fish do not feel pain. Anim. Sentience.

[43] Rose J.D., Arlinghaus R., Cooke S.J., Diggles B.K., Sawynok W., Stevens E.D., Wynne C.D. (2014). Can fish really feel pain?. Fish Fish..

[44] Cottee S.Y. (2010). Are fish the victims of ‘speciesism’? A discussion about fear, pain and animal consciousness. Fish Physiol. Biochem..

[45] Mather J.A. (2001). Animal suffering: An invertebrate perspective. J. Appl. Anim. Welf. Sci..

[46] Elwood R.W., Barr S., Patterson L. (2009). Pain and stress in crustaceans?. Appl. Anim. Behav. Sci..

[47] Aquiloni L., Gherardi F. (2008). Evidence of cryptic mate choice in crayfish. Biol. Lett..

[48] Bateson M., Desire S., Gartside S.E., Wright G.A. (2011). Agitated Honeybees Exhibit Pessimistic Cognitive Biases. Curr. Biol..

[49] Siegford J.M., Bernardo T.M., Malinowski R.P., Laughlin K., Zanella A.J. (2005). Integrating animal welfare into veterinary education: Using an online, interactive course. J. Vet. Med. Educ..

[50] World Organisation for Animal Health (OIE) (2012). Competencies of Graduating Veterinarians (‘Day 1 Graduates’) to Assure National Veterinary Services of Quality.

[51] Williams V.M. (2002). Conflicts of interest affecting the role of veterinarians in animal welfare. ANZCCART News.

[52] Paul E.S., Podberscek A.L. (2000). Veterinary education and students’ attitudes towards animal welfare. Vet. Rec..

[53] Williams V.M., Lascelles B.D., Robson M.C. (2005). Current attitudes to, and use of, peri-operative analgesia in dogs and cats by veterinarians in New Zealand. N. Z. Vet. J..

[54] Anil L., Anil S.S., Deen J. (2005). Pain detection and amelioration in animals on the farm: Issues and options. J. Appl. Anim. Welf. Sci..

[55] Heleski C.R., Mertig A.G., Zanella A.J. (2004). Assessing attitudes toward farm animal welfare: A national survey of animal science faculty members. J. Anim. Sci..

[56] Hewson C.J., Dohoo I.R., Lemke K.A., Barkema H.W. (2007). Canadian veterinarians’ use of analgesics in cattle, pigs, and horses in 2004 and 2005. Can. Vet. J..

[57] Whay H.R., Huxley J. (2005). Pain relief in cattle: A practitioner’s perspective. Cattle Pract..

[58] Levine E.D., Mills D.S., Houpt K.A. (2005). Attitudes of veterinary students at one US college toward factors relating to farm animal welfare. J. Vet. Med. Educ..

[59] Heleski C.R., Mertig A.G., Zanella A.J. (2006). Stakeholder attitudes toward farm animal welfare. Anthrozoös.

[60] Hellyer P.W., Frederick C., Lacy M., Salman M.D., Wagner A.E. (1999). Attitudes of veterinary medical students, house officers, clinical faculty, and staff toward pain management in animals. J. Am. Vet. Med. Assoc..

[61] Martin F., Ruby K., Farnum J. (2003). Importance of the human-animal bond for pre-veterinary, first-year, and fourth-year veterinary students in relation to their career choice. J. Vet. Med. Educ..

[62] Hazel S.J., Signal T.D., Taylor N. (2011). Can teaching veterinary and animal-science students about animal welfare affect their attitude toward animals and human-related empathy?. J. Vet. Med. Educ..

[63] Pollard-Williams S., Doyle R.E., Freire R. (2014). The influence of workplace learning on attitudes toward animal welfare in veterinary students. J. Vet. Med. Educ..

[64] Coleman G.J., McGregor M., Hemsworth P.H., Boyce J., Dowling S. (2003). The relationship between beliefs, attitudes and observed behaviours of abattoir personnel in the pig industry. Appl. Anim. Behav. Sci..

[65] Hemsworth P.H. (2003). Human–animal interactions in livestock production. Appl. Anim. Behav. Sci..

[66] Kauppinen T., Vainio A., Valros A., Rita H., Vesala K.M. (2010). Improving animal welfare: Qualitative and quantitative methodology in the study of farmers’ attitudes. Anim. Welf..

[67] Kauppinen T., Vesala K.M., Valros A. (2012). Farmer attitude toward improvement of animal welfare is correlated with piglet production parameters. Livest. Sci..

[68] Te Velde H.T., Aarts N., Van Woerkum C. (2002). Dealing with ambivalence: Farmers’ and consumers’ perceptions of animal welfare in livestock breeding. J. Agric. Environ. Ethics.

[69] Vanhonacker F., Verbeke W., Van Poucke E., Tuyttens F.A. (2008). Do citizens and farmers interpret the concept of farm animal welfare differently?. Livest. Sci..

[70] Hubbard C., Bourlakis M., Garrod G. (2007). Pig in the middle: Farmers and the delivery of farm animal welfare standards. Br. Food J..

[71] Bock B.B., van Huik M.M. (2007). Animal welfare: The attitudes and behaviour of European pig farmers. Br. Food J..

[72] Rushen J., de Passillé A.M., Munksgaard L. (1999). Fear of people by cows and effects on milk yield, behavior, and heart rate at milking. J. Dairy Sci..

[73] Coleman G.J., Hemsworth P.H., Hay M., Cox M. (2000). Modifying stockperson attitudes and behaviour towards pigs at a large commercial farm. Appl. Anim. Behav. Sci..

[74] Hemsworth P.H., Coleman G.J., Barnett J.L. (1994). Improving the attitude and behaviour of stockpersons towards pigs and the consequences on the behaviour and reproductive performance of commercial pigs. Appl. Anim. Behav. Sci..

[75] European Commission Special Eurobarometer 270, September–October 2006. http://ec.europa.eu/public_opinion/archives/ebs/ebs_270_en.pdf.

[76] Schröder M.J., McEachern M.G. (2014). Consumer value conflicts surrounding ethical food purchase decisions: A focus on animal welfare. Int. J. Consum. Stud..

[77] Harper G.C., Henson S. (2001). Consumer Concerns about Animal Welfare and the Impact on Food Choice.

[78] Knight S., Nunkoosing K., Vrij A., Cherryman J. (2003). Using grounded theory to examine people’s attitudes towards how animals are used. Soc. Anim..

[79] Miele M. Report Concerning Consumer Perceptions and Attitudes towards Farm Animal Welfare.

[80] Heleski C.R., Zanella A.J. (2006). Animal science student attitudes to farm animal welfare. Anthrozoös.

[81] European Commission (2016). Special Eurobarometer 442. http://data.europa.eu/euodp/en/data/dataset/S2096_84_4_442_ENG.

[82] Spooner J.M., Schuppli C.A., Fraser D. (2014). Attitudes of Canadian citizens toward farm animal welfare: A qualitative study. Livest. Sci..

[83] Mayfield L.E., Bennett R.M., Tranter R.B., Wooldridge M.J. (2007). Consumption of welfare-friends food products in Great Britain, Italy and Sweden, and how it may be influenced by consumer attitudes to, and behaviour towards, animal welfare attributes. Int. J. Soc. Food Agric..

[84] Hall C., Sandilands V. (2007). Public attitudes to the welfare of broiler chickens. Anim. Welf..

[85] Ngapo T.M., Dransfield E., Martin J.F., Magnusson M., Bredahl L., Nute G.R. (2004). Consumer perceptions: Pork and pig production. Insights from France, England, Sweden and Denmark. Meat Sci..

[86] McKendree M.G., Croney C.C., Widmar N.J. (2014). Effects of demographic factors and information sources on United States consumer perceptions of animal welfare. J. Anim. Sci..

[87] Tonsor G.T., Olynk N.J. (2011). Impacts of Animal Well-Being and Welfare Media on Meat Demand. J. Agric. Econ..

[88] Hazel S.J., O’Dwyer L., Ryan T. (2015). Chickens are a lot smarter than I originally thought: Changes in student attitudes to chickens following a chicken training class. Animals.

[89] Signal T.D., Taylor N. (2007). Attitude to animals and empathy: Comparing animal protection and general community samples. Anthrozoös.

[90] Bejaei M., Wiseman K., Cheng K.M. (2015). Developing logistic regression models using purchase attributes and demographics to predict the probability of purchases of regular and specialty eggs. Br. Poult. Sci..

[91] Coleman G. Public Perceptions of Animal Pain and Animal Welfare in Australian Animal Welfare Strategy.

[92] Frewer L.J., Kole A., Van de Kroon S.M., de Lauwere C. (2005). Consumer attitudes towards the development of animal-friendly husbandry systems. J. Agric. Environ. Ethics.

[93] European Commission Special Eurobarometer 229. http://ec.europa.eu/public_opinion/archives/ebs/ebs_229_en.pdf.

[94] Driscoll J.W. (1992). Attitudes towards animal use. Anthrozoös.

[95] Kellert S.R., Berry J.K. (1981). Knowledge, Affection and Basic Attitudes toward Animals in American Society.

[96] Knight S., Vrij A., Cherryman J., Nunkoosing K. (2004). Attitudes towards animal use and belief in animal mind. Anthrozoös.

[97] Kendall H.A., Lobao L.M., Sharp J. (2006). Public concern with animal well-being: Place, social structural location, and individual experience. Rural Sociol..

[98] Baron-Cohen S. (2003). The Essential Difference: Men, Women and the Extreme Male Brain.

[99] Jamieson J., Reiss M.J., Allen D., Asher L., Parker M.O., Wathes C.M., Abeyesinghe S.M. (2015). Adolescents care but don’t feel responsible for farm animal welfare. Soc. Anim..

[100] Eldridge J.J., Gluck J. (1996). Gender differences in attitudes toward animal research. Ethics Behav..

[101] Broida J., Tingley L., Kimball R., Miele J. (1993). Personality differences between pro-and anti-vivisectionists. Soc. Anim..

[102] Vanhonacker F., Verbeke W., van Poucke E., Tuyttens F.A. (2007). Segmentation based on consumers’ perceived importance and attitude toward animal welfare. Int. J. Soc. Food Agric..

[103] Herzog H.A., Betchart N.S., Pittman R.B. (1991). Gender, sex role orientation, and attitudes toward animals. Anthrozoös.

[104] Peek C.W., Dunham C.C., Dietz B.E. (1997). Gender, relational role orientation, and affinity for animal rights. Sex Roles.

[105] Adams C. (1990). The Sexual Politics of Meat: A Feminist Vegetarian Critical Theory.

[106] Rothgerber H. (2013). Real men don’t eat (vegetable) quiche: Masculinity and the justification of meat consumption. Psychol. Men Masculinity.

[107] Bowd A.D., Bowd A.C. (1989). Attitudes toward the treatment of animals: A study of Christian groups in Australia. Anthrozoös.

[108] Deemer D.R., Lobao L.M. (2011). Public concern with farm-animal welfare: Religion, politics, and human disadvantage in the food sector. Rural Sociol..

[109] Paul E.S., Serpell J.A. (1993). Childhood pet keeping and humane attitudes in young adulthood. Anim. Welf..

[110] Allport G.W. (1954). The Nature of Prejudice.

[111] Tawse J. (2010). Consumer attitudes towards farm animals and their welfare: A pig production case study. Biosci. Horizons.

[112] Singer P. (1975). Animal Liberation.

[113] Kagan S. (2016). What’s wrong with speciesism?. J. Appl. Philos..

[114] Singer P. (2016). Why speciesism is wrong: A response to Kagan. J. Appl. Philos..

[115] Phillips C.J., McCulloch S. (2005). Student attitudes on animal sentience and use of animals in society. J. Biol. Educ..

[116] Driscoll J.W. (1995). Attitudes towards animals: Species ratings. Soc. Anim..

[117] Bastian B., Loughnan S., Haslam N., Radke H.R. (2012). Don’t mind meat? The denial of mind to animals used for human consumption. Personal. Soc. Psychol. Bull..

[118] Prunty J., Apple K.J. (2013). Painfully aware: The effects of dissonance on attitudes toward factory farming. Anthrozoös.

[119] Loughnan S., Haslam N., Bastian B. (2010). The role of meat consumption in the denial of moral status and mind to meat animals. Appetite.

[120] De Boer J., Hoogland C.T., Boersema J.J. (2007). Towards more sustainable food choices: Value priorities and motivational orientations. Food Qual. Preference.

[121] De Backer C.J., Hudders L. (2015). Meat morals: Relationship between meat consumption consumer attitudes towards human and animal welfare and moral behavior. Meat Sci..

[122] Festinger L. (1957). A Theory of Cognitive Dissonance.

